# Influence of Polytetrafluoroethylene Content, Compaction Pressure, and Annealing Treatment on the Magnetic Properties of Iron-Based Soft Magnetic Composites

**DOI:** 10.3390/molecules29174019

**Published:** 2024-08-25

**Authors:** Mei Song, Fan Luo, Yajing Shang, Zhongxia Duan

**Affiliations:** 1 Key Laboratory of High Density Electromagnetic Power and Systems (Chinese Academy of Sciences), Institute of Electrical Engineering, Chinese Academy of Sciences, Haidian District, Beijing 100190, China; songmei@mail.iee.ac.cn (M.S.); luofan@mail.iee.ac.cn (F.L.); shangyajing@mail.iee.ac.cn (Y.S.); 2 Institute of Electrical Engineering and Advanced Electromagnetic Drive Technology, Qilu Zhongke, Jinan 250101, China; 3 University of Chinese Academy of Sciences, Shijingshan District, Beijing 100049, China

**Keywords:** soft magnetic composites, polytetrafluoroethylene, compaction pressure, annealing treatment, magnetic properties

## Abstract

To improve the magnetic properties of iron-based soft magnetic composites (SMCs), polytetrafluoroethylene (PTFE) with excellent heat resistance, electrical insulation, and extremely high electrical resistivity was chosen as an insulating coating material for the preparation of iron-based SMCs. The effects of PTFE content, compaction pressure, and annealing treatment on the magnetic properties of Fe/PTFE SMCs were investigated in detail. The results demonstrate that the PTFE insulating layer is successfully coated on the surface of iron powders, which effectively reduces the core loss, increases the resistivity, and improves the frequency stability and the quality factor. Under the combined effect of optimal PTFE content, compaction pressure, and annealing treatment, the iron-based SMCs exhibit a high effective permeability of 56, high saturation magnetization of 192.9 emu/g, and low total core losses of 355 mW/cm^3^ and 1705 mW/cm^3^ at 50 kHz for B_m_ = 50 mT and 100 mT. This work provides a novel insulating coating layer that optimizes magnetic properties and is advantageous for the development of iron-based SMCs. In addition, it also provides a comprehensive understanding of the relationship between process parameters and magnetic properties, which is of great guiding significance for scientific research and industrial production.

## 1. Introduction

Soft magnetic composites (SMCs) are obtained by using ferromagnetic powders as raw materials, coating the surface of the powders with an insulating dielectric layer, and then pressing the powders into a desired shape, followed by annealing treatment [1,2,3]. Because of their unique properties with high magnetic induction, three-dimensional magnetic isotropy, high magnetic permeability, low coercivity, and low core loss, SMCs have attracted considerable attention and are broadly applied in the field of electromagnetic devices such as inductors, filters, motors, and transformers [1,2,3,4,5].

Pure iron powder, as the most cost-effective, widely used, and promising soft magnetic material, has been employed for the preparation of SMCs [6,7,8,9]. The performance of iron-based SMCs is greatly affected by insulating layer properties [10,11,12,13,14] and manufacturing parameters such as compaction pressure [15,16] and annealing treatment [17,18,19]. In general, insulating materials include organics (such as epoxy resin [20], phenolic resin [14,21], silicone resins [22,23], and polyimide [24]) and inorganics (such as phosphates [25], oxides [26,27,28,29,30,31], and ferrite [32,33,34]). Organic-coated iron-based SMCs have the advantages of a simple preparation process, low cost, and excellent magnetic properties, and they are widely used in industrial production. Regarding traditional organic-coated iron-based SMCs, high pressure is required to compact the iron powders coated with an insulating phase into the designed shape. This process inevitably produces internal stresses and defects, causing an increase in magnetic loss. So, the annealing treatment process should be carried out to choose a high enough temperature that will maximally eliminate internal stresses and defects [35,36]. However, traditional organic insulation materials exhibit low heat-resistant temperatures and poor high-temperature stability. On the one hand, crystallization or decomposition may occur during high-temperature annealing [35,37], resulting in a sharp decrease in electrical resistivity and an increase in eddy current loss for iron-based SMCs. This limits the annealing temperature, which cannot effectively eliminate internal stresses, resulting in high hysteresis loss in iron-based SMCs. On the other hand, high operating temperatures can deteriorate organic insulation materials, thereby reducing the magnetic performance and service life of iron-based SMCs. Therefore, in order to ensure the effective use and long-term stability of iron-based SMCs, the operating temperature range of iron-based SMCs is limited to −65~125 °C. It is difficult to meet the needs of diverse engineering applications and complex service environments as well as high-efficiency, low-energy-consumption, high-frequency, and high-power applications for high-performance, high-reliability iron-based SMCs. Therefore, there is an urgent need to select new insulating coating material and optimize process parameters to prepare high-performance and high-reliability iron-based SMCs.

Polytetrafluoroethylene (PTFE) has been used as an irreplaceable material for various industrial applications. It has been widely used in heat-resistant materials, corrosion-resistant materials, insulation materials, and sealing materials because of its excellent chemical stability, corrosion resistance, heat resistance, sealing performance, electrical insulation, extremely high electrical resistivity, and good aging resistance [38,39,40,41]. PTFE shows high thermal stability up to 200~260 °C. As an insulation material, it is not only expected to maintain the integrity of the insulation layer while eliminating internal stresses and defects but it is also expected to enable SMCs to operate stably at higher temperatures. In addition, PTFE provides extremely high electrical resistivity, which can significantly reduce eddy current loss and improve the high-frequency magnetic performance of SMCs. Until now, there has been no report on iron-based SMCs prepared by utilizing a PTFE insulating layer.

In this paper, iron-based SMCs were prepared by utilizing PTFE as an insulating layer. The influences of PTFE content, compaction pressure, and annealing temperature on the magnetic properties of Fe/PTFE SMCs were discussed. The optimized process parameters and the performance control method were obtained by the analysis and discussion, which can provide guidance for regulating the properties of soft magnetic composites.

## 2. Results and Discussion

### 2.1. Characterization of the Fe/PTFE Composite Powders and Corresponding SMCs

**Figure 1:**
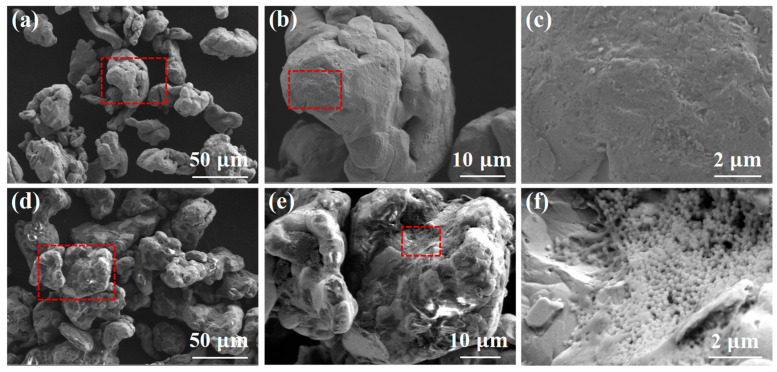
(**a**) SEM image of the raw iron powders; (**b**,**c**) are a partial enlargement corresponding to the red boxes in (**a**,**b**); (**d**) SEM image of the Fe/PTFE composite powders containing 3 wt% PTFE; (**e**,**f**) are a partial enlargement corresponding to the red boxes in (**d**,**e**).

The Fe/PTFE composite powders were fabricated by mixing raw iron powders and different amounts of PTFE powders (0 wt%, 1 wt%, 2 wt%, 3 wt%, 4 wt%, and 5 wt%) through ball milling (see Materials and Methods and Supplementary Materials for experimental details). Figure 1 shows the surface morphology of the raw iron powders and ball-milled Fe/PTFE composite powders containing 3 wt% PTFE at different magnifications. The raw iron particles exhibit relatively good dispersion and a smooth and clean surface (Figure 1a–c). The Fe/PTFE composite powders appear aggregated to some extent. The surface of the composite powders tends to become rougher and exhibits obvious floc aggregates. The floc aggregates are composed of small spherical particles (Figure 1d–f). These results confirm that a relatively thick insulating layer formed on the surface of the iron particles.

**Figure 2:**
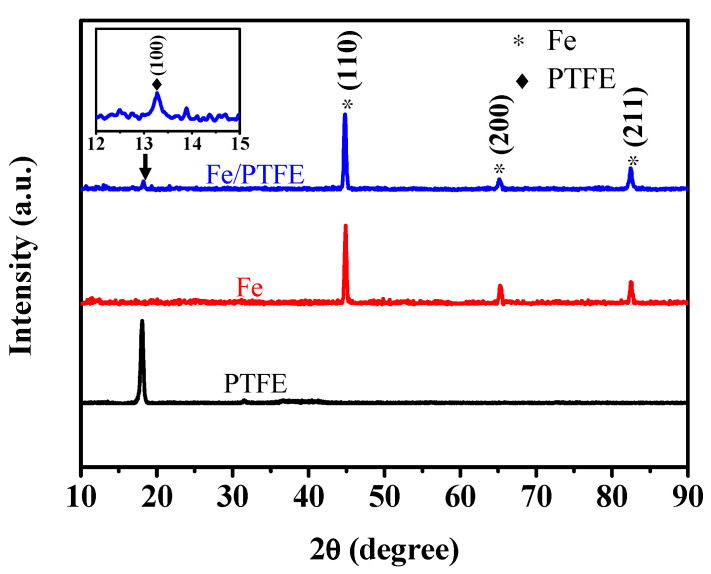
XRD patterns of the PTFE powders (black line), raw iron powders (red line), and Fe/PTFE composite powders containing 3 wt% PTFE (blue line).

To further confirm the phase structures of the insulating layer on the surface of the iron particles, an XRD pattern of the Fe/PTFE composite powders containing 3 wt% PTFE (blue line in Figure 2) was carried out. For comparison, XRD patterns of the raw iron powders (red line in Figure 2) and PTFE powders (black line in Figure 2) were also measured. As can be observed, the raw iron powders show three obvious characteristic peaks centered at 44.7° (110), 65.0° (200), and 82.3° (211), respectively, which correspond to the α-Fe phase (JCPDs Card: 99-0064). For the Fe/PTFE composite powders, a new characteristic peak occurs at 18.1° (100), which is consistent with the standard characteristic peak of PTFE (JCPDs Card: 54-1595). The appearance of the characteristic peak proves the presence of PTFE. Combined with SEM images of the Fe/PTFE composite powders containing 3 wt% PTFE (Figure 1d–f), these results confirm that a PTFE insulation layer is successfully coated on the surface of the iron particles.

**Figure 3:**
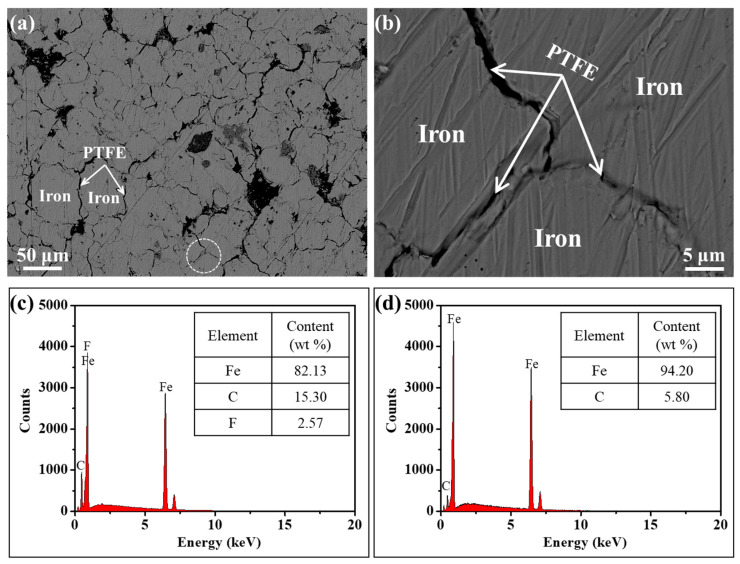
(**a**) SEM image of the cross-section of Fe/PTFE SMCs coated with 3 wt% PTFE; (**b**) is a partial enlargement corresponding to the white circle in (**a**); EDS spectra of (**c**) PTFE insulating layer and (**d**) iron particles for the Fe/PTFE SMCs coated with 3 wt% PTFE.

The Fe/PTFE composite powders containing 3 wt% PTFE were compacted at 648 MPa, followed by annealing at 150 °C for 90 min to obtain Fe/PTFE SMCs (see Materials and Methods and Supplementary Materials for experimental details). To investigate the microstructure, especially the distribution of the PTFE insulating layer in Fe/PTFE SMCs, back-scattered electron images of the cross-sectional structure of the Fe/PTFE SMCs coated with 3 wt% PTFE were carried out. Figure 3a gives the morphology of the cross- section for the Fe/PTFE SMCs coated with 3 wt% PTFE. Figure 3b is a partial magnification corresponding to the white circle in Figure 3a. It can be seen that the iron particles are separated from each other by a PTFE insulating layer. The corresponding EDS spectra of the PTFE insulating layer and iron particles for the Fe/PTFE SMCs coated with 3 wt% PTFE are shown in Figure 3c,d. It is clear that the iron content decreases while the C content increases and the F content appears at the interface between iron particles, which indicates that the surface of the iron particles is covered by a PTFE insulating layer.

### 2.2. Effect of PTFE Content on the Magnetic Properties of Fe/PTFE SMCs

**Figure 4:**
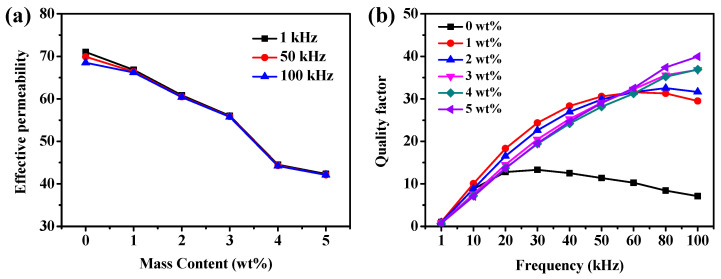
(**a**) Effective permeability of Fe/PTFE SMCs as a function of PTFE contents at three different frequencies; (**b**) quality factor of Fe/PTFE SMCs with different PTFE contents versus frequency.

The Fe SMCs coated with different PTFE contents (0 wt%, 1 wt%, 2 wt%, 3 wt%, 4 wt%, and 5 wt%) were prepared by compacting at 648 MPa, followed by annealing at 150 °C for 90 min. Figure 4a shows the effective permeability as a function of PTFE contents at three different frequencies. With the increase in PTFE contents from 0 wt% to 5 wt%, the effective permeability decreases gradually from 71.0 to 42.4 (a decrease of 40%) (Table 1). The main reasons are as follows (see Supplementary Materials for Equations (S1) and (S2)): on the one hand, with the increase in PTFE content, the thickness of the insulation layer on the surface of iron particles increases, thereby increasing the air gap between metal particles; on the other hand, the magnetic dilution by nonmagnetic PTFE will increase the internal demagnetization field in Fe SMCs and then weaken the magnetic interaction between the iron particles. However, the Fe SMCs uncoated with PTFE exhibit poor frequency stability subjected to the deteriorating effect of eddy currents. Specifically, as the frequency increases from 1 kHz to 100 kHz, the effective permeability decreases monotonically from 71.0 to 68.5 (a decrease of 4%) (Table 1). Regarding PTFE-coated SMCs, the frequency stability becomes better with the increased PTFE content. This should be because the increase in PTFE coating thickness and resistivity (Table 1) can effectively isolate the adjacent powders and reduce the eddy current. The quality factor is a parameter of the energy storage and consumption performance of SMCs during alternating magnetization. In general, the higher the quality factor on behalf of SMCs, the lower the rate of energy loss. Figure 4b shows the variation in quality factor with tested frequencies for Fe SMCs coated with different PTFE contents. The Fe/PTFE SMCs with 3–5 wt% PTFE exhibit a higher and more stable quality factor at higher frequencies (60–100 kHz), which may be attributed to sufficient PTFE to form a continuous isolating layer. Thus, the 3 wt% PTFE-coated Fe SMCs cause a lower decrease and better frequency stability in effective permeability and a higher and more stable quality factor compared to other Fe/PTFE SMCs.

**Figure 5:**
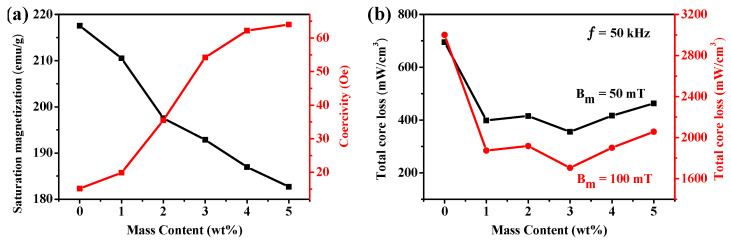
(**a**) Saturation magnetization (black line) and coercivity (red line) of Fe/PTFE SMCs containing different PTFE contents at a magnetic field of 20,000 Oe; (**b**) total core loss of Fe/PTFE SMCs with different PTFE contents measured at 50 kHz for B_m_ = 50 mT and 100 mT, respectively.

The static magnetic properties of the Fe SMCs coated with different PTFE contents were studied. As shown in Figure 5a and Table 1, the value of saturation magnetization in Fe/PTFE SMCs decreases from 217.6 emu/g to 182.7 emu/g with increasing PTFE contents from 0 wt% to 5 wt%; this decreasing saturation magnetization is attributed to the promoted magnetic dilution by the increasing nonmagnetic PTFE contents. However, the coercivity in Fe/PTFE SMCs increases from 15.2 Oe to 64 Oe, indicating an opposite tendency to the variation in saturation magnetization. This should be a consequence of the fact that more pinning sites induced by nonmagnetic PTFE suppress the movement of the magnetic domain walls [31].

Low core loss is required for soft magnetic materials for application in the field of electromagnetic devices. The total core loss ($P_{t}$) is calculated using the following equation [3,42,43,44]:

(1) $$P_{t}=P_{hy}+P_{ed}+P_{re}\approx K_{hy}Af+K_{ed}B^{2}f^{2}d^{2}/\rho$$

The total core loss ($P_{t}$) includes three parts: hysteresis loss ($P_{hy}$), eddy current loss ($P_{ed}$), and residual loss ($P_{re}$).$P_{re}$ is caused by the relaxation process in magnetization and can be ignored because it is much smaller than $P_{ed}$ and $P_{hy}$ [1,45]. $P_{hy}$ is known to be caused by hysteresis behavior and can be obtained from the corresponding hysteresis loop [46]. $P_{ed}$ is caused by an eddy current generated by an alternating current field [47]. Meanwhile, A, B, d, and ρ indicate the area of the DC–hysteresis loop, magnetic flux density, eddy current radius, and electrical resistivity, respectively. $K_{hy}$ and $K_{ed}$ are dimensional constants. According to Equation (1), the hysteresis loss is proportional to the area of the DC–hysteresis loop, and the eddy current loss is inversely proportional to the electrical resistivity of samples. Therefore, improving the soft magnetic DC properties will decrease the area of the DC–hysteresis loop and thus decrease the hysteresis loss. Meanwhile, the increase in resistivity will lead to a decrease in eddy current loss. Figure 5b shows the PTFE content dependence of the total core loss in Fe/PTFE SMCs measured at 50 mT and 100 mT, 50 kHz. As the PTFE content increases, the iron particles are isolated by a PTFE insulation layer, which increases the electrical resistivity in Fe SMCs (Table 1) and reduces their eddy currents. However, the higher level of PTFE content increases the demagnetizing field, which increases the coercivity and area of the DC–hysteresis loop, thereby increasing the hysteresis loss of samples. Therefore, the Fe/PTFE SMCs containing 3 wt% PTFE demonstrate the minimal total core losses of 355 mW/cm^3^ and 1705 mW/cm^3^ measured at 50 mT and 100 mT, 50 kHz (Figure 5b and Table 1), which exhibit a 49% and 43% reduction compared to the uncoated Fe SMCs.

Therefore, it is clear that the Fe/PTFE SMCs containing 3 wt% PTFE have a low core loss, high effective permeability, and high saturation magnetization, in comparison with other amounts of PTFE-insulated Fe/PTFE SMCs. As a result, it can be concluded that 3 wt% is a relatively ideal insulating content for the PTFE-coated iron-based soft magnetic composites. To further optimize the magnetic properties of Fe/PTFE SMCs, the effects of compaction pressure and annealing temperature on the magnetic properties of the Fe/PTFE SMCs containing 3 wt% PTFE were investigated.

### 2.3. Effect of Compaction Pressure on the Magnetic Properties of Fe/PTFE SMCs

**Figure 6:**
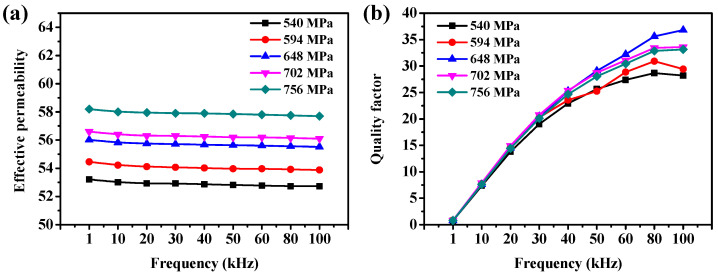
(**a**) Effective permeability and (**b**) quality factor of Fe/PTFE SMCs containing 3 wt% PTFE with various compaction pressures versus frequency.

The compaction pressure is one of the important factors affecting the magnetic properties of SMCs. To investigate the effect of compaction pressure on the magnetic properties of Fe/PTFE SMCs, the Fe/PTFE SMCs containing 3 wt% PTFE were prepared by pressing at various compaction pressures (540 MPa, 594 MPa, 648 MPa, 702 MPa, and 756 MPa), followed by annealing at 150 °C for 90 min. Figure 6a illustrates the effective permeability of Fe/PTFE SMCs at different compaction pressures. It can be found that effective permeability frequency dependence shows a similar tendency. In addition, the effective permeability of all Fe/PTFE SMCs at different compaction pressures decreases only slightly by about 9‰ with the frequency increases from 1 kHz to 100 kHz, confirming the excellent stability of the effective permeability over the entire frequency range. Meanwhile, the effective permeability of Fe/PTFE SMCs gradually increases from about 53 to 58 for all frequencies with the increasing pressure from 540 MPa to 756 MPa (Figure S1), which can be attributed to an increase in SMCs’ density (Table 2 and Equation (S3)), which then leads to a decrease in the internal air gap and an increase in the volume fraction of the magnetic materials. Figure 6b shows the quality factor of Fe/PTFE SMCs prepared at various compaction pressures. It is clear that the quality factor of the Fe/PTFE SMCs compacted at 648 MPa is highest and most stable at higher frequencies (50–100 kHz). As the compaction pressure further increases, cracks appear on the surface of the particles and the damage to the integrity of the insulation layer leads to a deterioration of the quality factor.

**Figure 7:**
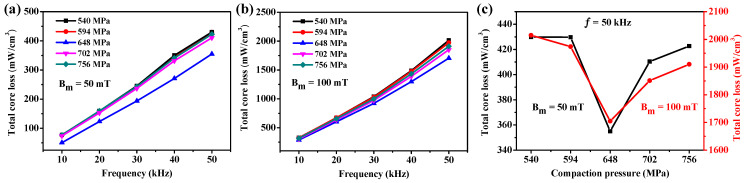
(**a**,**b**) Total core loss as a function of frequency for the Fe/PTFE SMCs containing 3 wt% PTFE at different pressures measured at (**a**) B_m_ = 50 mT and (**b**) 100 mT; (**c**) total core loss as a function of compaction pressure for the Fe/PTFE SMCs containing 3 wt% PTFE measured at 50 kHz for B_m_ = 50 mT and 100 mT, respectively.

The effect of compaction pressure on the total core loss of Fe/PTFE SMCs is shown in Figure 7. According to Equation (1), the hysteresis loss is proportional to frequency and the eddy current loss is proportional to the square of frequency. Therefore, the total core loss of all Fe/PTFE SMCs at different compaction pressures significantly increases with increasing frequency, as shown in Figure 7a,b. The hysteresis loss is mainly dependent on the hysteresis behavior. The internal air gap between the magnetic particles can hinder the magnetization process of SMCs, which would decrease with the increase in density of SMCs. Therefore, the hysteresis loss decreases with the increase in compaction pressure from 540 MPa to 648 MPa. The eddy current loss mainly depends on the resistivity of Fe/PTFE SMCs at the same frequency. The higher the resistivity, the lower the eddy current loss. According to Table 2, gradually increased resistivity can be formed to reduce the eddy current loss for Fe/PTFE SMCs compacted from 540 MPa to 648 MPa. But excessive compaction pressure may create cracks in the surface of the particles, thus destroying the integrity of PTFE insulating layers and decreasing the resistivity between particles, which increases the eddy current loss [16]. Meanwhile, higher compaction pressure increases the residual stress, which increases the hysteresis loss. Therefore, the total core loss of Fe/PTFE SMCs decreases from 430 mW/cm^3^ to 355 mW/cm^3^ (50 mT, 50 kHz) and from 2015 mW/cm^3^ to 1705 mW/cm^3^ (100 mT, 50 kHz) when the compaction pressure increases from 540 MPa to 648 MPa and then begins to increase with a further increase in the compaction pressure (Figure 7c). Comparing these results, the optimum compaction pressure is 648 MPa.

### 2.4. Effect of Annealing Treatment on the Magnetic Properties of Fe/PTFE SMCs

**Figure 8:**
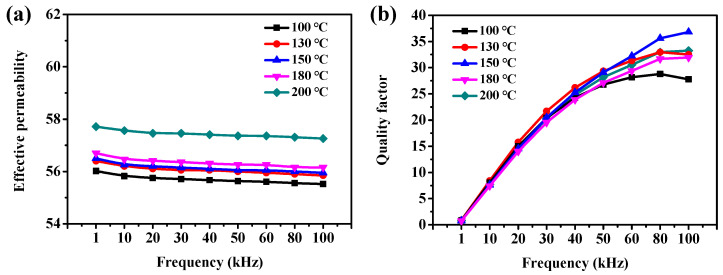
(**a**) Effective permeability and (**b**) quality factor as a function of frequency for the Fe/PTFE SMCs containing 3 wt% PTFE annealed at different temperatures.

Annealing treatment can eliminate the defects and residual stress inside SMCs, which has a significant effect on the magnetic properties of SMCs. The Fe/PTFE SMCs containing 3 wt% PTFE were prepared by pressing at 648 Mpa, followed by annealing at 100–200 °C for 90 min. Figure 8a illustrates the frequency-dependent effective permeability of Fe/PTFE SMCs at different annealed temperatures. The effective permeability of all samples shows excellent stability over the entire frequency range with a slight decrease of about 8‰ as the frequency increases from 1 kHz to 100 kHz at different temperatures. Meanwhile, the effective permeability of Fe/PTFE SMCs increases gradually as the temperature increases from 100 °C to 200 °C and reaches its highest value of about 58 (Figure S2). This result is because the effective permeability of Fe/PTFE SMCs is inversely proportional to the residual stress (Equation (S4)). As the temperature increases from 100 °C to 200 °C, the residual stress gradually decreases. In addition, the enhanced grain growth as well as interparticle bonding also result in a gradual increment in effective permeability.

**Figure 9:**
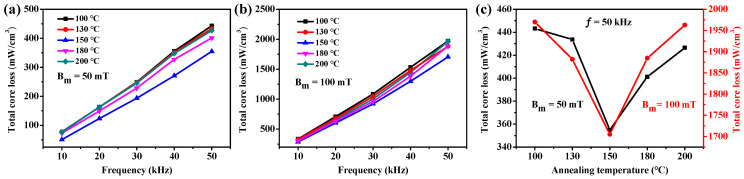
(**a**,**b**) Total core loss as a function of frequency for the Fe/PTFE SMCs containing 3 wt% PTFE annealed at different temperatures measured at (**a**) B_m_ = 50 mT and (**b**) 100 mT; (**c**) total core loss as a function of annealing temperature for the Fe/PTFE SMCs containing 3 wt% PTFE measured at 50 kHz for B_m_ = 50 mT and 100 mT, respectively.

Figure 9 shows the total core loss of Fe/PTFE SMCs annealed at different temperatures. The total core loss of all samples increases sharply with increasing frequencies. Also, the total core loss gradually decreases from 443 mW/cm^3^ to 355 mW/cm^3^ (50 mT, 50 kHz) and from 1970 mW/cm^3^ to 1705 mW/cm^3^ (100 mT, 50 kHz) with increasing annealing temperature from 100 °C to 150 °C. As the annealing temperature further increases from 150 °C to 200 °C, the total core loss dramatically increases to 427 mW/cm^3^ (50 mT, 50 kHz) and 1963 mW/cm^3^ (100 mT, 50 kHz), respectively. When annealed at lower temperatures (below 150 °C), the increase in annealing temperature releases the internal stress caused by the rapid pressing process, which decreases the total core loss. However, the further increase in annealing temperature above 150 °C results in the insulation layer being broken, which leads to a decrease in resistivity, thus increasing the total core loss. At the same time, the quality factor reaches its highest value at higher frequencies (50–100 kHz) for the Fe/PTFE SMCs annealed at 150 °C. When the annealing temperature exceeds 150 °C, the broken insulation layer leads to a decrease in quality factor (Figure 8b). Therefore, a higher annealing temperature (~150 °C) is considered to be the optimal heat treatment for the Fe/PTFE SMCs.

Based on all the results shown above, the Fe/PTFE SMCs exhibit a high effective permeability of 56, high saturation magnetization of 192.9 emu/g, and low total core losses of 355 mW/cm^3^ (50 mT, 50 kHz) and 1705 mW/cm^3^ (100 mT, 50 kHz) at the optimum PTFE content of 3 wt% for the Fe/PTFE SMCs compacted at 648 Mpa, followed by annealing at 150 °C for 90 min. The effect of coating content and manufacturing parameters on the magnetic properties of iron-based SMCs has been studied previously in a number of works. For example, Sun and colleagues [24] investigated the effect of polyimide content and curing temperature on the magnetic properties for iron-based SMCs and found that a low core loss of 780 W/Kg (measured at 50 mT and 100 kHz) and higher maximum permeability of 305 are achieved at the optimized polyimide content of 1.5 wt% and curing temperature of 400 °C. However, the magnetic flux density of iron-based SMCs coated with a polyimide insulating layer could only reach 1.20 T, which is 26% lower than that in our work. Very recently, by investigating the effect of MnO_2_ content on the microstructure and magnetic properties of iron-based SMCs, Zhang et al. [31] disclosed that the total core loss decreases gradually to 327.6 W/kg (50 kHz, 20 mT) when the MnO_2_ content increases to 10.0 wt%. However, the saturation magnetization of 180 A·m^2^/kg and magnetic permeability of 22 are much lower than those in our work. Wu and colleagues [15] investigated the effect of compress pressure on the magnetic properties of iron-based SMCs with a parylene insulating layer. The results indicated that the parylene C-coated iron-based SMCs have a lower value of hysteresis loss coefficient and eddy current coefficient. However, the magnetic induction value increases to 1.01 T at the optimized pressure of 650 MPa, which is 38% lower than that in our work. Therefore, our work provides progress and guidance for the research and development of high-performance iron-based SMCs.

## 3. Materials and Methods

### 3.1. Materials

Raw reduced iron powders (purity > 99.0%) provided by Chaoyang Jinhe Powder Metallurgy Material Co., Ltd. (Chaoyang, China) with a powder size of ≤75 µm were used as the matrix material. PTFE powders with an average particle size of 25 µm, supplied by Shanghai Aladdin Biochemical Technology Co., Ltd. (Shanghai, China), were used as the insulating coating material.

### 3.2. Preparation of the Fe/PTFE SMCs

The iron powders and different amounts of PTFE powders (0 wt%, 1 wt%, 2 wt%, 3 wt%, 4 wt%, and 5 wt%, respectively) were mixed by ball milling, with a total of 16 g of the mixture. The obtained Fe/PTFE composite powders were compacted in a toroid die at 648 MPa. Finally, the obtained ring specimens were annealed at 150 °C for 90 min. The optimal PTFE content was determined to be 3 wt%. To investigate the effect of compaction pressure, the ball-milled Fe/PTFE composite powders containing 3 wt% PTFE were compacted in a toroid die at 540 MPa, 594 MPa, 648 MPa, 702 MPa, and 756 MPa, respectively. Finally, the obtained ring specimens were annealed at 150 °C for 90 min. To explore the effect of annealing temperature, the ball-milled Fe/PTFE composite powders containing 3 wt% PTFE were compacted at 648 MPa into ring specimens. The batch of ring specimens was then annealed at 100 °C, 130 °C, 150 °C, 180 °C, and 200 °C for 90 min, respectively. Finally, the ball-milling time was 60 min and the rotational speed was 150 r/min. All compacted ring specimens had dimensions of an outer diameter of 20 mm, an inner diameter of 10 mm, and a height of 5 mm.

### 3.3. Characterizations

Surface topography of the raw iron powders, Fe/PTFE composite powders and Fe/PTFE SMCs, and corresponding EDS spectra were investigated by scanning electron microscopy (SEM, Supra 35VP, ZEISS, Jena, Germany) equipped with an energy-dispersive X-ray spectrometer (EDS, IE350PentaFetx-3, Oxford, UK). The phase structures of the raw PTFE powders, raw iron powders, and Fe/PTFE composite powders were characterized by X-ray diffraction (XRD, D500, Siemens, Germany) using Cu Kα radiation with a step size of 0.02° from 2θ = 10° to 90°. Effective permeability and quality factor of Fe/PTFE SMCs were obtained by an impedance analyzer (LCR, IM3570A988-06, HIOKI E.E. CORPORATION, Hioki, Japan) with a frequency range of 1 kHz to 100 kHz at a voltage of 300 mV with ring specimens winded by 20 turns of enameled wire. The static magnetic properties (including the saturation magnetization and coercivity) of Fe/PTFE SMCs were measured with a SQUID-VSM test system (Quantum Design, State of California, USA) with an applied magnetic field of 20,000 Oe at room temperature. The SMCs’ density was examined at least 3 times via the principle of Archimedes and the average value was recorded. The electrical resistivity of Fe/PTFE SMCs was tested via a 4-probe method (4-Point Probes Resistivity Measurement System, RTS-8, 4 Probes Technology Ltd., Guangzhou, China). The total core losses of Fe/PTFE SMCs were measured using a B–H curve analyzer (SY-8218, IWATSU, Tokyo, Japan) in a frequency ambit of 10–50 kHz and with a magnetic excitation level of 50 mT and 100 mT.

## 4. Conclusions

In this work, PTFE with excellent heat resistance, electrical insulation, and extremely high electrical resistivity was chosen to coat the iron powders. After adopting the compaction and annealing treatment processes, Fe/PTFE SMCs with a low core loss, high resistivity, high effective permeability, good frequency stability, high quality factor, and high saturation magnetization were finally prepared. The effects of PTFE content, compaction pressure, and annealing treatment on the magnetic properties of Fe/PTFE SMCs were investigated in detail. Results indicate that the low core losses of 355 mW/cm^3^ (50 mT, 50 kHz) and 1705 mW/cm^3^ (100 mT, 50 kHz), high effective permeability of 56, and high saturation magnetization of 192.9 emu/g were achieved at the optimum PTFE content of 3 wt% for the iron-based SMCs compacted at 648 Mpa, followed by annealing at 150 °C for 90 min.

## Figures and Tables

**Table 1:** Effective permeability, saturation magnetization, coercivity, total core loss, and resistivity of Fe/PTFE SMCs containing different PTFE contents.

PTFE Contents (wt%)	Effective Permeability		Saturation Magnetization (emu/g)	Coercivity (Oe)	Total Core Loss (mW/cm^3^)		Resistivity (mΩ·m)
					B_m_ = 50 mT	B_m_ = 100 mT	
	1 kHz	100 kHz			50 kHz	50 kHz	
0	71.0	68.5	217.6	15.2	695.1	3001	0.4
1	66.8	66.2	210.5	19.9	398.5	1874	4.3
2	60.8	60.4	197.5	35.5	415.4	1918	4.5
3	56.0	55.8	192.9	54.2	355	1705	19.9
4	44.5	44.2	187.0	62.2	416.5	1901	25.8
5	42.4	42.1	182.7	64	463.1	2057	97.3

**Table 2:** Density and resistivity of Fe/PTFE SMCs containing 3 wt% PTFE with various compaction pressures.

Compaction Pressure (MPa)	Density (g/cm^3^)	Resistivity (mΩ·m)
540	6.53	9.4
594	6.61	12.9
648	6.74	19.9
702	6.79	10.8
756	6.85	7.4

## Data Availability

The original contributions presented in this study are included in this article and Supplementary Materials. Further inquiries can be directed to the corresponding authors.

## Supplementary Materials

The following supporting information can be downloaded at: https://www.mdpi.com/article/10.3390/molecules29174019/s1, Figure S1: Effective permeability as a function of compaction pressure for the Fe/PTFE SMCs containing 3 wt% PTFE at different frequencies; Figure S2: Effective permeability as a function of annealing temperature for the Fe/PTFE SMCs containing 3 wt% PTFE at different frequencies. References [48,49,50,51,52] are cited in the Supplementary Materials.
