# Supplementary material for: Influence of Polytetrafluoroethylene Content, Compaction Pressure, and Annealing Treatment on the Magnetic Properties of Iron-Based Soft Magnetic Composites
Source: Molecules. 2024 Aug 25;29(17):4019. doi: 10.3390/molecules29174019 (PMC11396742; doi:10.3390/molecules29174019)
Supplement: Supplementary file 1 [file molecules-29-04019-s001.zip › molecules-3102723-supplementary.pdf]

Supplementary Information

Influence of polytetrafluoroethylene content, compaction pressure and annealing treatment on the magnetic properties of iron-based soft magnetic composites

Mei Song [1,2], Fan Luo [1,2], Yajing Shang [1,2] and Zhongxia Duan [1,2,3,*]

Section A: Experimental details

The ball milling of a mixture of iron powders and PTFE powders was performed using a QM-QX model omnidirectional planetary ball mill (Nanjing Nanda Instrument Co., Ltd., Nanjing, China). The compaction of the Fe/PTFE composite powders was operated using FW-4A powder compressing machine (Tianjin Tianguang Optics Instrument Co., Ltd., Tianjin, China). The pressed ring specimens were annealed using a blast drying box (Shanghai Fuma Experiment Equipment Co., Ltd., Shanghai, China).

Section B: Supporting figures

In general, the relationship between effective permeability (μ_e) and air gap between metal particles [48,49], volume fraction of the insulating coating material [50], SMCs' density [51], residual stress [52], is shown as follows:

$$\mu_e = \frac{D\mu_i}{D+\delta\mu_i} \tag{S1}$$

where D represents the diameter of the metal particles, μ_i is the intrinsic permeability of metal particles, δ is the air gap between metal particles.

$$\mu_e = \frac{3+(\mu_i-1)(3-3g)}{3+g(\mu_i-1)} \tag{S2}$$

where μ_i is the intrinsic permeability of metal particles, g is the volume fraction of the insulating coating material.

$$\mu_e = \frac{\mu_i m\rho'+2\mu_i m'\rho}{\mu_i m\rho'-(\mu_i-3)m'\rho} \tag{S3}$$

where μ_i is the intrinsic permeability of metal particles, m and ρ are the mass and density of the SMCs, m' and ρ' are the mass and density of the metal magnetic powders.

$$\mu_e = \frac{M_s^2}{\alpha K+\beta\lambda_s\sigma} \tag{S4}$$

where M_s is the saturation magnetization, α and β are constants, K is the magnetocrystalline anisotropy constant, λ_s is the magnetostrictive coefficient, and σ is the residual stress.

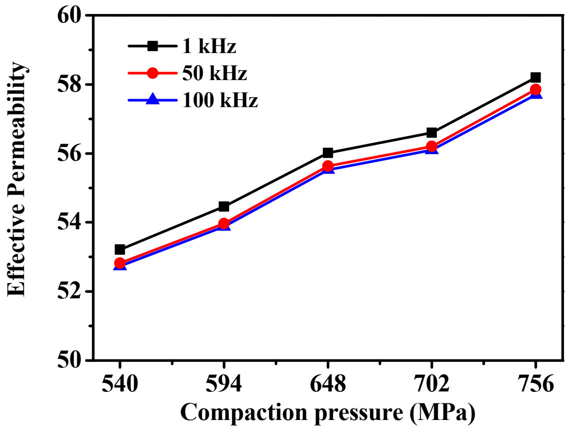

Figure S1. Effective permeability as a function of compaction pressure for the Fe/PTFE SMCs containing 3 wt% PTFE at different frequencies.

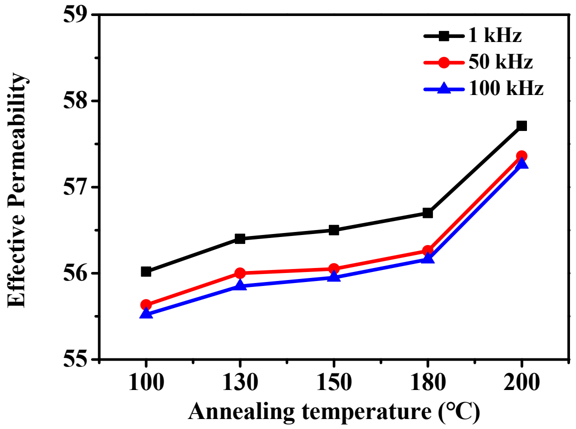

Figure S2. Effective permeability as a function of annealing temperature for the Fe/PTFE SMCs containing 3 wt% PTFE at different frequencies.
